# Rethinking EDSS-based ambulation assessment in multiple sclerosis using continuous variable monitoring

**DOI:** 10.1186/s42466-026-00501-8

**Published:** 2026-06-12

**Authors:** Noah M. Werner, Melanie Schuette, Ramona Hagler, Jan Voth, Balázs Danajka, Patricia Kirschner, Markus Heibel, Tjalf Ziemssen, Uwe K. Zettl, Sven G. Meuth, Marc Pawlitzki, Lars Masanneck

**Affiliations:** 1 Department of Neurology, Medical Faculty and University Hospital Düsseldorf, Heinrich-Heine University, Düsseldorf, Germany; 2 Hasso Plattner Institute, University of Potsdam, Potsdam, Germany; 3 Sauerlandklinik, Hachen, Germany; 4 Department of Neurology, Faculty of Medicine and University Hospital Carl Gustav Carus, Center of Clinical Neuroscience, Dresden, Germany; 5 Department of Neurology, Neuroimmunological Section, Rostock University Medical Center, Rostock, Germany

**Keywords:** Digital health technologies, Expanded disability status scale, Real-world mobility, Multiple sclerosis, Proof-of-concept study

## Abstract

**Background:**

Multiple sclerosis (MS) is a chronic inflammatory disease of the central nervous system (CNS) characterized by relapses and progressive disability. The Expanded Disability Status Scale (EDSS), used to quantify disability, is based on single, discretely assessed and potentially inaccurate patient-estimated walking ability, whereas digital health technologies (DHTs) enable continuous activity monitoring and more objective assessment of real-world functional performance.

**Methods:**

In this prospective observational study conducted at two German centers, patients with relapsing-remitting MS (RRMS) underwent clinical assessments at baseline (V1) and study completion (V2). Walking distance and step counts were measured using a measuring wheel and pedometer, while continuous physical activity was assessed via smartwatch-derived metrics.

**Results:**

Sixteen patients with RRMS were included (median age 57.5 years [interquartile range (IQR) 49.25–63.25]; median EDSS 4.5 [IQR 3.5–6]). Patient-estimated walking distance at V1 showed moderate correlation with clinically measured distance (Spearman‘s ρ = 0.60, *p* = 0.013), with 12 of 16 patients misjudging distances relative to EDSS thresholds. Walking distance showed intra-individual variability between V1 and V2 (median absolute difference: 113.6 m). Median daily walking distance (ρ = −0.61, *p* = 0.0123), step count (ρ = −0.64, *p* = 0.0082), and peak steps (ρ = −0.69, *p* = 0.0032) correlated negatively with EDSS.

**Conclusion:**

Patient-estimated maximum walking distance demonstrated moderate agreement with clinical performance and frequently crossed EDSS thresholds, while clinical assessments varied substantially within individuals over the short study duration, underscoring the limitations of single evaluations. In contrast, smartwatch-derived metrics aligned with clinical measures, reflected EDSS scores, and captured real-world mobility.

**Supplementary Information:**

The online version contains supplementary material available at 10.1186/s42466-026-00501-8.

## Introduction

Multiple Sclerosis (MS) is a chronic inflammatory disease of the central nervous system (CNS) [1, 2], characterized by both relapses and progressive disability accumulation [1, 3, 4, 5]. Despite substantial progress in reducing relapse rates in MS, many patients continue to accumulate disability, largely driven by progression independent of relapse activity (PIRA), which has emerged as a central determinant of long-term outcomes [6]. Various strategies have been proposed to characterize PIRA [8–10]. Nonetheless, the Expanded Disability Status Scale (EDSS) remains the primary clinical instrument to operationalize progression in MS in real-world practice [9, 11]. Despite its widespread use, the EDSS has been increasingly questioned [12]. It is a non-linear [13], predominantly motor-focused [12] scale, in which walking distance becomes a decisive determinant, particularly at scores between 4 and 6 [14]. Importantly, walking ability in MS can be conceptualized at various levels, including patients’ subjective perception of their walking capacity, the clinically assessed maximum walking distance under standardized conditions, and actual walking performance in everyday life. The reliance on patient-estimated walking capacity in routine clinical practice is often problematic, as many individuals are unable to accurately estimate their walking distance, thereby introducing considerable variability into the assessment [15]. In clinical practice, however, assessing maximum walking distance is time-consuming and is therefore often estimated based on patient report. Consequently, it remains uncertain to what extent EDSS values obtained during routine clinical visits truly reflect disease progression, given that everyday factors can also substantially influence the score [16].

To overcome these gaps, digital health technologies (DHTs), such as wearable devices, are becoming increasingly prevalent [17, 18] and are being used more frequently in neurological clinical trials [19], including studies in Duchenne muscular dystrophy [20], Parkinson’s disease [21], and Chronic Inflammatory Demyelinating Polyneuropathy [22, 23]. Recent studies in MS [24] further highlight the potential of DHTs, demonstrating that wearable-based monitoring can provide novel insights, with metrics such as average daily step count serving as additional indicators of disability severity and patient-specific characteristics [25]. Moreover, DHTs represent a promising solution for overcoming existing limitations by continuously collecting real-life data, including physical activity, sleep, and cognition [19]. Given the unprecedented longitudinal monitoring of physical activity that is made possible by these approaches, these DHTs may enable better detection of subtle fluctuations, particularly in early MS or mild progression, by continuously tracking day-to-day activity.

Accordingly, this study aimed to examine three key aspects of walking ability assessment in MS. First, we wanted to quantify the discrepancies between patient-estimated and clinically measured maximum walking distance and step count at the start of the study, including misclassification across EDSS-relevant walking thresholds. Second, we assessed short-term within-person variability of clinically measured maximum walking distance over a period of seven days. Finally, we investigated the relationships between EDSS, clinical walking performance, and smartwatch-derived real-world mobility metrics, with a particular focus on bout-based measures as indicators of walking ability in everyday life.

## Methods

### Study design

**Fig. 1:**
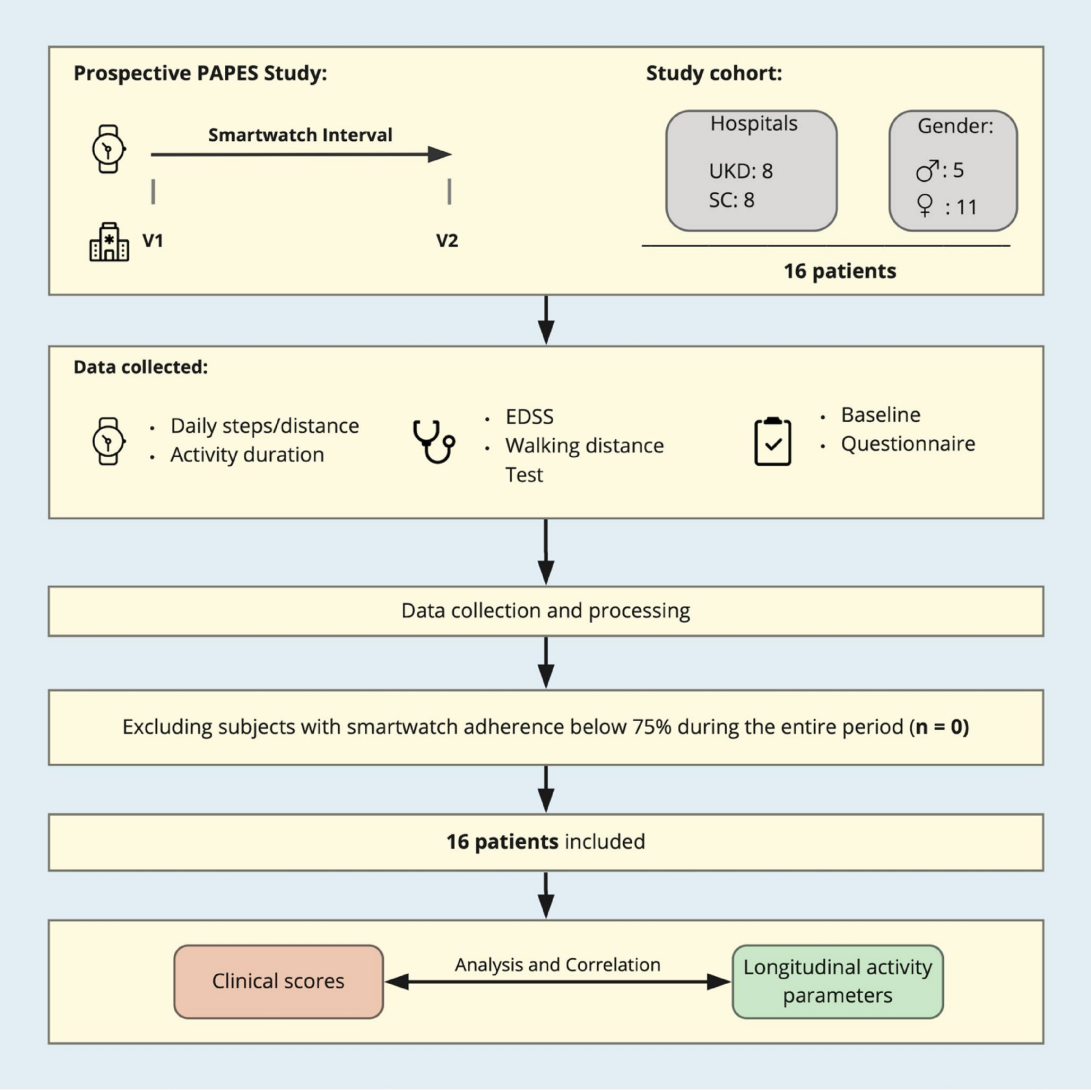
Study and Analysis Overview. This figure illustrates the design and process of the study, including patient recruitment at two centers, screening with inclusion and exclusion criteria, and subsequent study visits. The timeline depicts the study interval with two main assessment points. At each visit, clinical examinations and patient-reported outcome measures (questionnaires) were performed, including neurological disability scoring. The diagram further highlights the number of patients screened, excluded, and finally included in the analysis. Abbreviations: UKD, University Hospital Düsseldorf; SC, Sauerlandklinik Hachen; EDSS, Expanded Disability Status Scale

This prospective, two-center observational study was conducted at the Department of Neurology, University Hospital Düsseldorf, and Sauerlandklinik Hachen, Germany. Key inclusion criteria were age ≥ 18 years, a diagnosis of relapsing-remitting MS (RRMS) according to the revised 2017 McDonald criteria [26]. A clinically confirmed relapse within the preceding 90 days served as an exclusion criterion. After providing written informed consent, patients were equipped with a smartwatch (ScanWatch, Withings, Issy-les-Moulineaux, France) and instructed to wear it continuously, day and night, for the entire study period, enabling uninterrupted 24/7 data collection. Patients completed two scheduled study visits, including a walking distance test, at baseline (V1) and during the second study visit (V2) (for the study design, see Fig. 1). To better reflect everyday-life conditions, the interval between V1 and V2 was, whenever feasible, arranged to include a weekend.

Before performing the walking distance test, patients completed a questionnaire estimating the maximum distance they could walk before requiring a first break and the approximate number of steps this would involve (see Supplementary Fig. 1 in Online Resource 1). After completing the questionnaires and estimating their maximum walking distance and step count, patients were instructed to walk as far as possible. The exact start and end times were documented. Walking distance and steps were assessed using a digital measuring wheel (DigiRollerPlus 2, Calculated Industries) and a manual pedometer, while the smartwatch simultaneously recorded steps and distance digitally. The test was continued until the patients could no longer walk and required a break to define the maximum walking distance. During V2, the procedure was repeated without smartwatch recording.

### Outcome parameters

Disability status was evaluated using the EDSS [11]. The scale ranges from 0 (no disability) to 10 (death due to MS) [11]. In this study, we used the EDSS scores from previous medical examinations, which were not older than 30 days, for baseline characteristics, and did not perform a full EDSS assessment at V1 and V2. These days, we conducted the walking tests independently. All physicians in this study, as well as those who performed the EDSS, were certified EDSS raters.

During both study visits, the distance covered in meters and the number of steps were counted and documented on the test sheet provided. For the following analysis, the walking distances from V1 and V2 were evaluated comparatively.

### Digital measures

The smartwatch was used to record the following longitudinal activity parameters: daily steps as well as the estimated duration of soft (light), moderate, and intense (vigorous) activity (in seconds), according to the Physical Activity Guidelines for Americans [27] and the Compendium of Physical Activities [28]. In addition to the specified steps, we also recorded the algorithmically estimated distances covered in meters, as calculated by the smartwatch.

In order to approximate the maximum uninterrupted walking distance in everyday life, as assessed in the EDSS, we derived time-based activity intervals for steps and distance from the continuous smartwatch data. For the analysis, a walking interval was selected that was preceded by at least 15 min of inactivity and followed by another 15 min of inactivity. This ensured that the segment under consideration represented a complete, uninterrupted walking phase. Across the defined intervals, the longest step and distance streaks were extracted and summarized using minimum, maximum, and median values.

In addition, we used daily peak-step data from the smartwatch, which represents the highest step intensity achieved in a one-hour time window per day. The peak value is calculated by forming a rolling sum of the step count over a time window and then determining the maximum of this rolling sum. The returned peak-steps are the daily maximum step counts in such a 1-hour window during the observation period.

### Adherence calculation and criteria

An adherence of at least 75% of wearing time during waking hours (6:00 a.m. to 11:00 p.m.) was defined for the activity analysis. If there were days that were below this threshold, or if the individual adherence of the respective patients was not met on more than 25% of the days, they were excluded from the analysis. Adherence per patient is visualized in Supplementary Fig. 2 (in Online Resource 1). These criteria are comparable to the adherence criteria of our previous work [22].

### Software

Data analysis and visualization were computed using R (version 4.4.1) [29] in RStudio (version 2024.09.1 + 394), including the Base and Stats packages and ggplot2 [30]. Data handling and visualization were conducted using the tidyverse package [31], including dplyr and tidyr. Additional statistical analyses were supported by the Hmisc package [32]. A web application to synchronize, organize, and visualize data was set up based on the Dash framework version 2.6.1.

### Descriptive statistics and calculation of distribution characteristics

All analyses were exploratory in nature due to the sample size (*n* = 16) and the number of assessed correlations. Distributional assumptions were evaluated using the Shapiro–Wilk tests, in conjunction with inspection of summary distributions. Continuous variables are reported as mean (standard deviation, SD) when approximately normally distributed, and as median (interquartile range, IQR) otherwise.

Normality was assessed for all variables. Correlations were then examined using Pearson’s r for normally distributed continuous data, and Spearman’s rank correlation for non-normally distributed or ordinal data. We treated the analyses as exploratory and performed them without correction for multiple testing. As a secondary sensitivity analysis for analyses assessing associations between EDSS scores and corresponding patient-reported, smartwatch-derived, and V1 walking measures, FDR correction was applied using the Benjamini–Hochberg procedure to account for multiple comparisons.

For within-subject comparisons (V1 vs. V2) and comparisons of observed vs. estimated values at V1 and V2, paired t-tests were applied to normally distributed data, whereas Wilcoxon tests were used for non-normally distributed (non-parametric) data. Absolute differences between estimated and observed walking distances at V1 and between observed walking distances at V1 and V2 were summarized descriptively and tested against zero using one-sample t-tests or Wilcoxon signed-rank tests, depending on normality assessed by the Shapiro–Wilk test.

Given the exploratory nature of the analyses, results were primarily interpreted based on effect sizes and direction of associations, with p-values considered descriptive rather than confirmatory.

### Data availability statement

The data that support the findings of this study are available from the corresponding author upon reasonable request. Access may be subject to applicable ethical, legal, and institutional restrictions.

## Results

### Cohort characteristics

16 patients with RRMS were recruited across both centres. The median age at enrolment was 57.5 (IQR: 49.25–63.25) years, with a median disease duration of 12.5 (IQR: 3.50–22.00) years. Most patients (68.75%) were female. The baseline clinical values were a median EDSS of 4.5 (IQR: 3.0–6.0) and a median Body-Mass-Index (BMI) of 26.37 (IQR: 22.95–29.96). Of the sixteen patients, eleven received anti-CD20 treatment, one patient received teriflunomide, and four patients did not receive any specific disease-modifying therapy (see Supplementary Table 1 in Online Resource 1).

### Analysis of absolute deviations between patient-estimated and clinically measured walking distance and step counts at V1

**Fig. 2:**
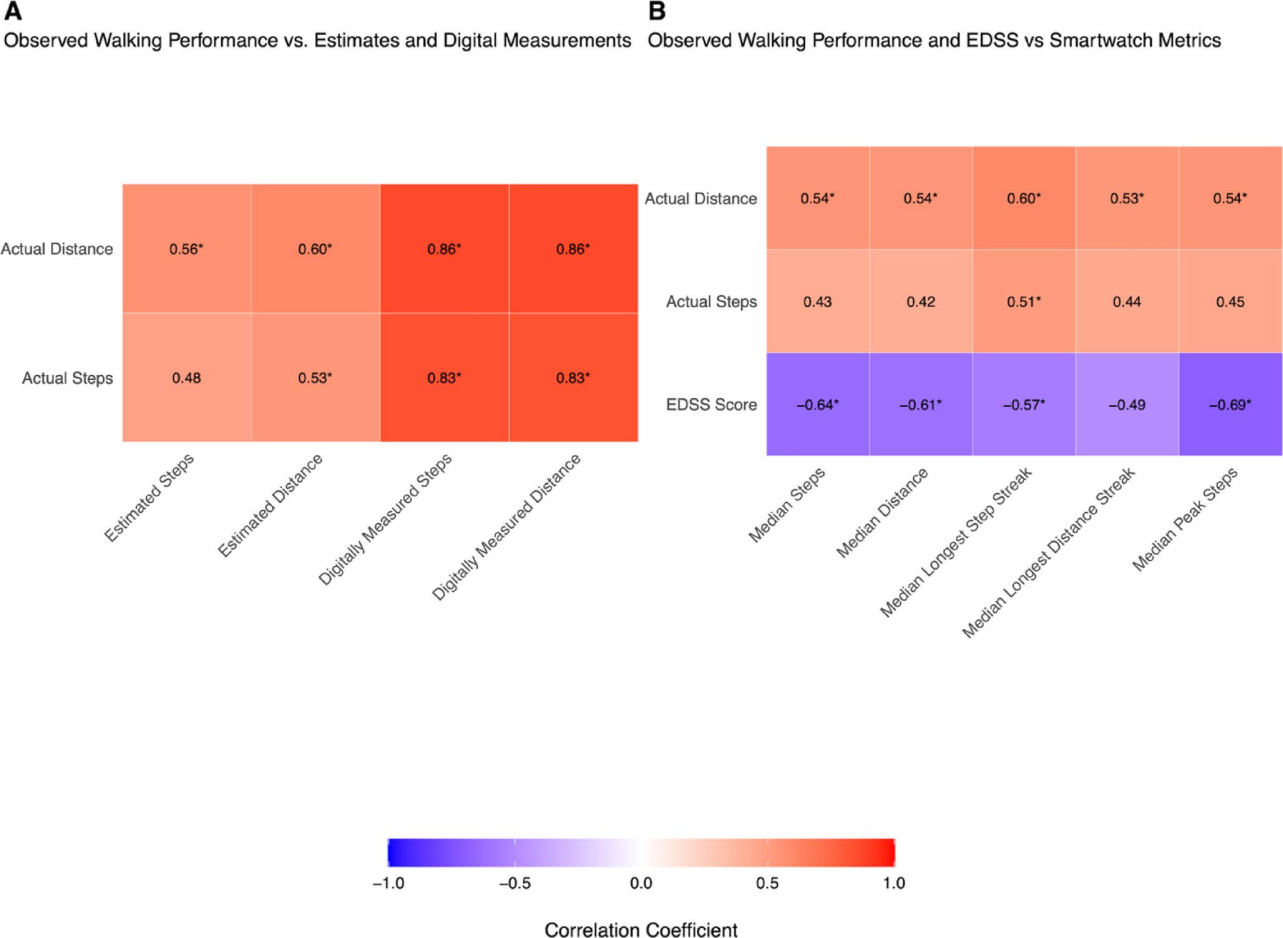
Correlation Heatmaps of observed Walking Performance, Digital Measurements, and Disability Scores. (**A**) Correlation between observed walking performance (actual distance and steps during the walking test) and patient-reported estimates, as well as digitally measured steps and distance. (**B**) Correlation between observed walking performance and EDSS scores with smartwatch-derived activity parameters, including median steps, median distance, longest step and distance streaks, and peak steps. Red indicates positive correlations, while blue indicates negative correlations. Asterisks denote statistically significant correlations (*p < 0.05). Pearson or Spearman correlation coefficients were applied depending on data distribution. Abbreviations: EDSS, Expanded Disability Status Scale

The median patient-estimated number of steps during the walking distance test in V1 was 700 (IQR: 137.50–1400), and the median patient-estimated walking distance was 400 m (IQR: 62.50–600). The median number of steps measured by the pedometer during the walking distance test in V1 was 443 steps (IQR: 229.50–1255.50). The median walking distance measured by the measuring wheel during the same test was 214.33 m (IQR: 111.80–694.58). The patient-estimated steps showed a moderate positive, but not significant, correlation with the steps measured by the pedometer in the V1 walking distance test (Spearman’s ρ = 0.48, *p* = 0.059). The patient-estimated walking distance correlated moderately and significantly with the distance measured using the measuring wheel in the V1 walking distance test (ρ = 0.60, *p* = 0.013; Fig. 2) and remained statistically significant after FDR correction (Supplementary Table 2; Supplementary Fig. 3 in Online Resource 1).

**Fig. 3:**
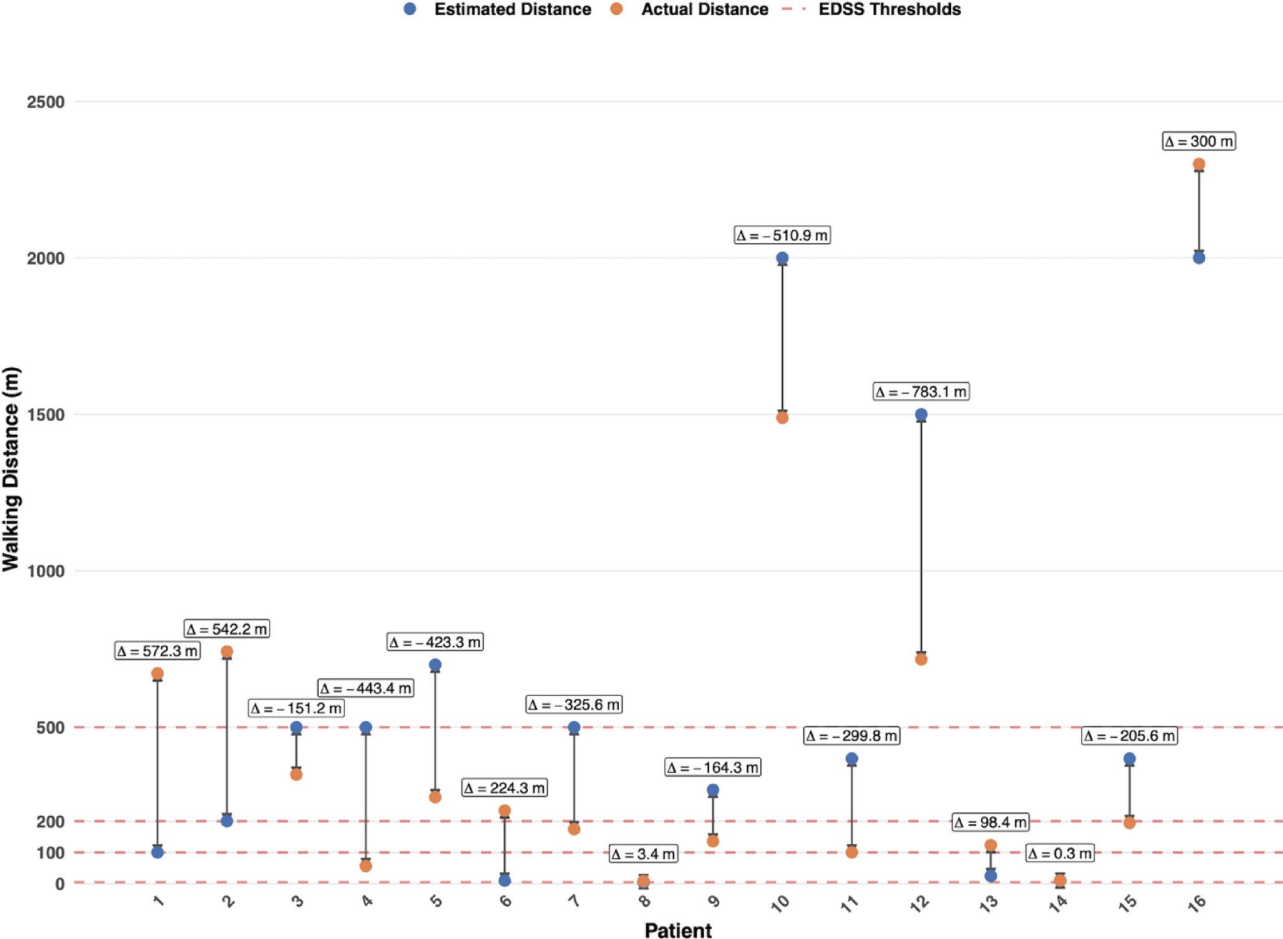
Comparison of Estimated vs. Actual Walking Distance. Scatter plots illustrate individual patient performance in the walking test. Estimated values (blue) before attending and actual values (orange) of the performed walking distance are shown. The x-axis depicts individual study participants, while the y-axis represents the actual walking distance in meters achieved. Red dashed lines indicate clinically relevant thresholds of walking performance (100 m, 200 m, 500 m). Abbreviations: EDSS, Expanded Disability Status Scale

Of note, 12 of 16 patients misclassified their walking distance relative to EDSS-defined thresholds (Fig. 3; Supplementary Table 3 in Online Resource 1).

### Analysis of clinically measured walking distance at V1 and V2

**Fig. 4:**
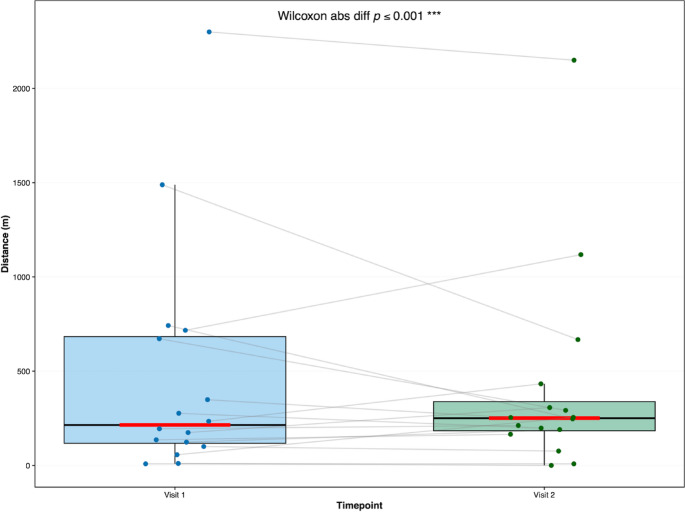
Comparison of walking distance between Visit 1 and Visit 2. Boxplots display the distribution of walking distances at visit 1 (blue) and visit 2 (green). Individual patient trajectories are connected with grey lines. Red bars indicate the mean value for each group. Substantial within-person variability is observed, with changes occurring in both directions. Absolute differences were significantly greater than zero (*p* < 0.001), indicating pronounced intraindividual variability

Over a median interval of seven days (IQR: 7–10), clinically measured maximum walking distance showed substantial within-person variability (median absolute difference 113.6 m; Fig. 4; Supplementary Tables 4–5 in Online Resource 1), with changes occurring in both directions. Comparing the group median of the measured walking distance in V1, the measured distance in V2 was slightly higher at 250.69 m (IQR: 177.63–369.81).

In V1, the Spearman correlation between patient-estimated walking distances and EDSS was ρ = -0.90 (*p* < 0.001), while clinically measured walking distance correlated moderately with EDSS (ρ = -0.47, *p* = 0.064). In V2, the Spearman correlation between patient-estimated walking distances and EDSS was ρ = -0.54 (*p* = 0.029), while clinically measured walking distance was more strongly associated with EDSS (ρ = -0.61, *p* = 0.013).

### Smartwatch analysis

Overall, smartwatch adherence during predefined waking hours was high, with most participants meeting the prespecified threshold for inclusion in activity analyses (see Supplementary Fig. 2 in Online Resource 1). Daily physical activity was assessed based on the number of steps taken and distance walked, measured by the smartwatch. Across recorded days, median daily step count was 2210 (IQR 796.50–4118.50) and median daily smartwatch-derived walking distance was 1779.8 m (IQR 590.30–3136.80). The distribution of daily steps is shown in Supplementary Fig. 4 (in Online Resource 1).

### Correlation of smartwatch-derived activity metrics with EDSS scores

**Fig. 5:**
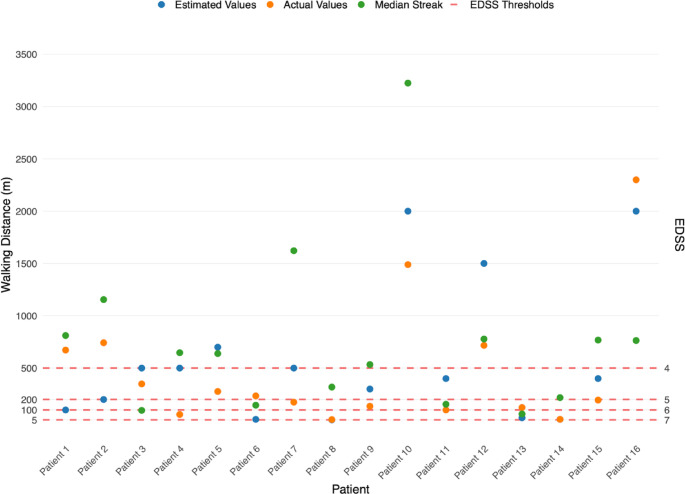
Comparison of Estimated, Clinical Measured and Median longest Distance Streak. Scatter plots illustrate individual patient walking performance. Estimated walking distances (blue) prior to the study visit, actual walking distances (orange), and median longest distance streaks measured via digital monitoring (green) are displayed for each participant. The x-axis represents individual study participants, while the y-axis shows walking distance in meters. Red dashed lines indicate clinically relevant thresholds of walking performance (5 m, 100 m, 200 m, 500 m). Abbreviations: EDSS, Expanded Disability Status Scale

Smartwatch-derived step counts and distances during the V1 walking distance test correlated strongly with clinically measured values (steps: ρ = 0.83, *p* < 0.001; distance: ρ = 0.86, *p* < 0.001; Fig. 2), remaining statistically significant after FDR correction (Supplementary Table 2; Supplementary Fig. 3 in Online Resource 1). The median values for the longest steps and distance streak in everyday life per patient, derived from the smartwatch recordings, correlated moderately and significantly with the clinical measurements at V1(steps: ρ = 0.51, *p* = 0.0464; distance: ρ = 0.53, *p* = 0.0373; Fig. 2), although these associations did not remain significant after FDR correction (Supplementary Table 2; Supplementary Fig. 3 in Online Resource 1). A graph of the distance streak derived by the smartwatch, together with the patient-estimated distances and those measured clinically, is shown in Fig. 5.

With regard to EDSS scores, both the median walking distance and the median number of steps per subject show a moderate negative correlation when using smartwatch-derived everyday data (ρ = -0.61, *p* = 0.0123 for walking distance; ρ = -0.64, *p* = 0.0082 for steps), as does the median peak-steps per subject (ρ = -0.69, *p* = 0.0032) during the entire period in which the smartwatch was worn (Fig. 2), remaining statistically significant after FDR correction (Supplementary Table 2; Supplementary Fig. 3 in Online Resource 1).

## Discussion

In this proof-of-concept study, we connected patient-estimated, clinically assessed, and smartwatch-derived walking measures in people with MS. Three observations stand out: self-estimated walking distance showed only moderate agreement with clinical performance and frequently crossed EDSS-relevant ambulation thresholds; clinically measured walking distance varied substantially within individuals over just seven days; and smartwatch-derived measures correlated closely with reference measurements during in-clinic testing and likely captured complementary aspects of real-world mobility over a 7-day period.

This study demonstrated a strong correlation between clinically assessed and digitally measured walking distance and step count. This close agreement provided the basis for linking everyday activity parameters with functional clinical measures. At the same time, many patients substantially misclassified their own physical performance. Despite the overall correlation between in-clinic testing and patient-estimated maximum walking distance, discrepancies in patients‘ estimates were large enough in several cases to result in EDSS-relevant shifts, affecting 12 of 16 patients in our cohort. These findings are consistent with previous reports showing that both patients and physicians frequently misjudge walking distance [15, 33].

Consistent with these observations, studies in which daily activity was objectively measured using smartwatches have shown that patient-estimated walking distance correlates only weakly with objectively measured performance, with most patients underestimating their walking ability, leading to clinically relevant EDSS misclassification [34]. This discrepancy between subjective perception and objectively measured performance has previously been described [35–38] and is also reflected in patient reports of perceived disability despite stable EDSS scores [39]. Since motor impairment plays a central role in MS disability assessment, especially in the mid-range of the scale (EDSS 4.0–6.0) [14], even small variations in walking distance can lead to a change in disability grade.

A second important observation from our study was the short-term variability in clinically measured walking distances. Even between two clinical assessments separated by only a median of seven days, maximum walking distances showed considerable fluctuations. Although the group median changed only slightly, the individual fluctuations showed that even frequent clinical tests capture only a snapshot of walking performance and may not take into account short-term fluctuations in motor function. Even subtle deterioration of the disease can contribute to this variability. It has been shown that insidious progression occurs even at lower EDSS scores and often goes unnoticed in patients who are officially classified as having RRMS [7]. Such gradual changes are easily overlooked in sporadic clinical examinations, underscoring the need for complementary tools that can more reliably capture fluctuations in practice.

In consideration of these limitations, DHTs offer a promising alternative. Previous studies have shown that accelerometer-based activity tracking correlates with EDSS [40, 41] and several groups have investigated the relationship between step counts and clinical scores [42]. The median values of the peak-steps also showed a strong correlation with the EDSS, offering the possibility of approaching as closely as possible the individual limit of the maximum possible walking distance before a resting period and thus showing the potential as a short-term marker. By analyzing predefined walking-distance intervals in our data, we likewise observed the expected day-to-day fluctuations but were able to derive daily or weekly medians. These aggregated measurements correlated with clinical walking tests and provided a more stable representation of typical walking ability than isolated clinical assessments, again underscoring the potential of objective measurements in real-world conditions. Even when walking distance is measured according to strict standardized procedures for EDSS assessments, performance in MS patients is likely influenced by prior daily activity. Fatigue severity in MS increases throughout the day, typically peaking in the late afternoon [43], and physical exertion further exacerbates fatigue [44], potentially impairing walking performance in later assessments, highlighting the need for more frequent monitoring, which DHTs may facilitate. The high adherence to smartwatch use observed in this study, consistent with our previous long-term trials [22], indicates that patients are willing to wear such devices on a daily basis.

This study has several limitations. EDSS ratings were performed by different physicians, potentially introducing inter-rater variability; however, this reflects usual clinical practice in clinical settings, and in addition, all raters were certified EDSS raters with extensive clinical expertise. Furthermore, we did not incorporate the patient-reported walking distance from V1 into the current EDSS assessment because the EDSS was not recalculated if the prior score was less than 30 days old. Overall, patient-reported walking distance and EDSS-derived ambulatory status were closely aligned, suggesting good concordance with the contemporaneous EDSS estimate even without updating the score based on V1. Minor discrepancies between V1 walking-distance estimates and those documented at the time of EDSS determination were observed in a small number of cases, further underscoring that self-estimated walking distance can fluctuate even over short time intervals. Furthermore, the walking distance assessments were not standardized for the time of day, which may have led to deviations due to daily fluctuations. However, this is in line with standard clinical practice and highlights a limitation that often occurs with single-point assessments. Additionally, due to the proof-of-concept design, our cohort included patients with comparatively higher EDSS scores. Future studies should evaluate larger and more heterogeneous cohorts, including individuals with lower disability levels and longer follow-up, to validate our findings and further explore the potential of digital health technologies to detect subtle disease progression at earlier stages of MS. Given the small cohort size, the present findings should be interpreted as exploratory. Nevertheless, we applied FDR correction to correlations between real-world parameters and EDSS, as well as to clinical assessments of the V1 walking test. The sensitivity analysis using FDR correction showed overall similar trends, with the main findings remaining consistent after adjustment. However, larger cohorts are needed to confirm these observations.

Most consumer wearable devices use accelerometer-based technology to detect walking activity [45], as does the Withings Scanwatch. Established frameworks for validating wearable-based biometric monitoring technologies, such as the V3 framework (verification, analytical validation, clinical validation), have been proposed [46]. However, to our knowledge, no peer-reviewed validation study has specifically evaluated the step count or walking distance algorithms of the Withings ScanWatch. While older Withings models have been validated, these findings cannot be directly extrapolated to the ScanWatch [47–49]. In the present study, smartwatch-recorded data showed good correlation with manual measurements (measuring wheel, pedometer) during V1. This provides preliminary analytical verification; however, confirmation in larger cohorts is warranted. There is wide variability in step count accuracy based on device model, movement speed, and testing environment, and the gold standard remains video-recorded step count [45]. External influences such as infections or mobility aid use can confound activity signals [50, 51].

In our analysis, a 15-minute period of inactivity before and after each episode was used to define self-initiated walking bouts that may represent a complete, uninterrupted walking phase. To contextualize this choice, alternative interval lengths (5, 10, 20, and 30 min) were explored. Shorter intervals may be more susceptible to short-term fluctuations and therefore less representative, whereas longer intervals may be too prolonged to adequately capture spontaneous daily mobility. However, these alternative intervals were not included in the final analysis and were therefore not systematically evaluated. Consequently, no definitive conclusions can be drawn regarding the optimal duration of inactivity intervals, as this was not the primary focus of the study. Nevertheless, our findings suggest that bout-based measures provide a feasible approach for assessing real-world daily activity. Future studies with larger cohorts and longer monitoring periods are needed to systematically evaluate different interval thresholds and to determine the most appropriate definition of real-world walking bouts. In contrast to the six-minute walking test, which requires rest without specifying a fixed duration [52, 53], the 15-minute inactivity threshold was pragmatically chosen to distinguish isolated walking from continuous activity. However, bout-detection algorithms in free-living accelerometry studies vary considerably [54], and the sensitivity of our results to this threshold was not formally evaluated.

In conclusion, patient-estimated walking distance reflected EDSS-related disability only partially and may be unreliable around clinically relevant ambulation thresholds. Short-term variability in clinical walking distance tests further limits the interpretation of single snapshot assessments. Therefore, even if the maximum walking distance would be measured in everyday clinical practice despite the high time expenditure, the values would be of limited usefulness. Smartwatch-derived real-world mobility metrics, particularly bout-based measures such as peak-steps and longest streaks, offer a feasible complementary perspective on ambulatory function and may help contextualize EDSS-focused assessments in routine care and research. Nevertheless, smartwatches, such as those owned by patients, are of course not regulated and also have various differences. This makes it all the more important to always check all parameters in terms of quality. In our study setting, it was easy to track patient adherence over 7 days using the same smartwatch, and adherence was consistently high. Over a longer period, it may be advisable to check these parameters regularly to avoid snapshots and ensure continuous monitoring, as well as to take into account the different manufacturer standards. Integrating validated digital mobility parameters into routine care may improve detection of disease progression, offer the potential to reduce reliance on time-consuming walking tests, and enable a more differentiated, patient-centered assessment of disability in multiple sclerosis.

## Electronic Supplementary Material

Supplementary Material 1.

## Data Availability

The datasets used and/or analyzed during the current study are available from the corresponding author on reasonable request. Due to data protection regulations and patient confidentiality, the data are not publicly available.

## Abbreviations

MS: Multiple sclerosis
CNS: Central nervous system
PIRA: Progression independent of relapse activity
EDSS: Expanded disability status scale
DHTs: Digital health technologies
RRMS: Relapsing-remitting MS
V1: Visit 1
V2: Visit 2
IQR: Interquartile range
SD: Standard deviation
BMI: Body-mass-index
m: Meter

## References

[1] Portaccio, E., Magyari, M., Havrdova, E. K., Ruet, A., Brochet, B., & Scalfari, A. (2024). u. a. Multiple sclerosis: emerging epidemiological trends and redefining the clinical course. *Lancet Reg Health - Eur September*, *44*, 100977. 10.1016/j.lanepe.2024.100977 10.1016/j.lanepe.2024.100977 PMC11496978 39444703

[2] Weinshenker, B. G. (1995). The Natural History of Multiple Sclerosis. *Neurol Clin 1 Februar*, *13*(1), 119–146. 10.1016/S0733-8619(18)30064-1 7739500

[3] Jakimovski, D., Bittner, S., Zivadinov, R., Morrow, S. A., Benedict, R. H., & Zipp, F. (2024). u. a. Multiple sclerosis. *The Lancet Januar*, *403*(10422), 183–202. 10.1016/S0140-6736(23)01473-3 10.1016/S0140-6736(23)01473-3 37949093

[4] Katz Sand, I. (2015). Classification, diagnosis, and differential diagnosis of multiple sclerosis. *Curr Opin Neurol Juni*, *28*(3), 193–205. 10.1097/WCO.0000000000000206 10.1097/WCO.0000000000000206 25887774

[5] Madill, E., Healy, B. C., Molazadeh, N., Polgar-Turcsanyi, M., Glanz, B. I., & Weiner, H. L. (2024). u. a. Serum glial fibrillary acidic protein predicts disease progression in multiple sclerosis. *Ann Clin Transl Neurol Oktober*, *11*(10), 2719–2730. 10.1002/acn3.52187 10.1002/acn3.52187 PMC11514927 39238198

[6] Portaccio, E., Bellinvia, A., Fonderico, M., Pastò, L., Razzolini, L., & Totaro, R. (2022). u. a. Progression is independent of relapse activity in early multiple sclerosis: a real-life cohort study. *Brain 27 August*, *145*(8), 2796–2805. 10.1093/brain/awac111 10.1093/brain/awac111 35325059

[7] University of California, San Francisco, M. S. E. P. I. C., Team, Cree, B. A. C., Hollenbach, J. A., Bove, R., Kirkish, G., & Sacco, S. (2019). u. a. Silent progression in disease activity–free relapsing multiple sclerosis. *Ann Neurol Mai*, *85*(5), 653–666. 10.1002/ana.25463 10.1002/ana.25463 PMC6518998 30851128

[8] Alonso, R., Casas, M., Rojas, J. I., Eizaguirre, M. B., Lazaro, L., & Pita, C. (2025). u. a. Progression independent of relapse activity (PIRA) in the Era of high-efficacy treatments. *Mult Scler Relat Disord Dezember*, *104*, 106775. 10.1016/j.msard.2025.106775 10.1016/j.msard.2025.106775 41005018

[9] Müller, J., Cagol, A., Lorscheider, J., Tsagkas, C., Benkert, P., & Yaldizli, Ö. (2023). u. a. Harmonizing Definitions for Progression Independent of Relapse Activity in Multiple Sclerosis: A Systematic Review. *JAMA Neurol 1 November*, *80*(11), 1232. 10.1001/jamaneurol.2023.3331 10.1001/jamaneurol.2023.3331 37782515

[10] Bouweriks, D., Kreiter, D., Knippenberg, S. A. M., Damoiseaux, J. G. M. C., Smolders, J., & Van Eijk (2026). JJJ, u. a. MSpine: a prospective longitudinal study of spinal cord lesions in multiple sclerosis: MRI monitoring and prognostic factors for active disease. A study protocol. *Neurol Res Pract 17 April*, *8*(1), 23. 10.1186/s42466-026-00465-9 10.1186/s42466-026-00465-9 PMC13091239 41998718

[11] Kurtzke, J. F. (1983). Rating neurologic impairment in multiple sclerosis: an expanded disability status scale (EDSS). *Neurology November*, *33*(11), 1444–1452. 10.1212/wnl.33.11.1444. Located at: Medline; 6685237. 10.1212/wnl.33.11.1444 6685237

[12] Kappos, L., D’Souza, M., Lechner-Scott, J., & Lienert, C. (2015). Mai. On the origin of Neurostatus. Mult Scler Relat Disord. 20150411. Aufl. ;4(3):182–5. Located at: Medline; 26008933. 10.1016/j.msard.2015.04.001 10.1016/j.msard.2015.04.001 26008933

[13] Twork, S., Wiesmeth, S., Spindler, M., Wirtz, M., Schipper, S., & Pöhlau, D. (2010). u. a. Disability status and quality of life in multiple sclerosis: non-linearity of the Expanded Disability Status Scale (EDSS). *Health Qual Life Outcomes Dezember*, *8*(1), 55. 10.1186/1477-7525-8-55 10.1186/1477-7525-8-55 PMC2890700 20529265

[14] Ringel, I., & Zettl, U. K. (2006). Estimates of the walking distance in multiple sclerosis patients and their effect on the EDSS. *J Neurol Mai*, *253*(5), 666–667. 10.1007/s00415-006-0049-7 10.1007/s00415-006-0049-7 16619120

[15] Sharrack, B., & Hughes, R. A. C. (1997). Reliability of distance estimation by doctors and patients: cross sectional study. *BMJ 20 Dezember*, *315*(7123), 1652–1654. 10.1136/bmj.315.7123.1652 10.1136/bmj.315.7123.1652 PMC2128034 9448528

[16] Albrecht, H., Wotzel, C., Erasmus, L. P., Kleinpeter, M., Konig, N., & Pollmann, W. (2001). April. Day-to-day variability of maximum walking distance in MS patients can mislead to relevant changes in the Expanded Disability Status Scale (EDSS): average walking speed is a more constant parameter. Mult Scler. ;7(2):105–9. Located at: Medline; 11424630. 10.1177/135245850100700206 10.1177/135245850100700206 11424630

[17] Feldhege, F., Mau-Moeller, A., Lindner, T., Hein, A., Markschies, A., & Zettl, U. (2015). u. a. Accuracy of a Custom Physical Activity and Knee Angle Measurement Sensor System for Patients with Neuromuscular Disorders and Gait Abnormalities. *Sensors 6 Mai*, *15*(5), 10734–10752. 10.3390/s150510734 10.3390/s150510734 PMC4482003 25954954

[18] Marra, C., Chen, J. L., Coravos, A., & Stern, A. D. (2020). Quantifying the use of connected digital products in clinical research. *Npj Digit Med 3 April*, *3*(1), 50. 10.1038/s41746-020-0259-x 10.1038/s41746-020-0259-x PMC7125096 32285011

[19] Masanneck, L., Gieseler, P., Gordon, W. J., Meuth, S. G., & Stern, A. D. (2023). Februar. Evidence from ClinicalTrials.gov on the growth of Digital Health Technologies in neurology trials. NPJ Digit Med. 20230210. Aufl. 10. ;6(1):23. Located at: PubMed-not-MEDLINE; 36765123. 10.1038/s41746-023-00767-1 10.1038/s41746-023-00767-1 PMC9918454 36765123

[20] Servais, L., Camino, E., Clement, A., McDonald, C. M., Lukawy, J., & Lowes, L. P. (2021). u. a. First Regulatory Qualification of a Novel Digital Endpoint in Duchenne Muscular Dystrophy: A Multi-Stakeholder Perspective on the Impact for Patients and for Drug Development in Neuromuscular Diseases. *Digit Biomark 5 August*, *5*(2), 183–190. 10.1159/000517411 10.1159/000517411 PMC8460979 34723071

[21] Von Below, D., Wallerstedt, S. M., & Bergquist, F. (2026). Wearable sensor measurements in relation to clinical characteristics and mortality in patients with Parkinson’s disease. *Neurol Res Pract 11 März*, *8*(1), 14. 10.1186/s42466-026-00474-8 10.1186/s42466-026-00474-8 PMC12980861 41814450

[22] Masanneck, L., Voth, J., Gmahl, N., Jendretzky, K., Huntemann, N., & Werner, N. M. u. a. Digital Activity Markers in Chronic Inflammatory Demyelinating Polyneuropathy. *Annals Of Clinical And Translational Neurology*. 9. Juli 2025;acn3.70137. 10.1002/acn3.70137 10.1002/acn3.70137 PMC12516250 40635238

[23] Voth, J., Von Gall, C., Gmahl, N., Werner, N. M., Hörste, G. M. Z., & Meuth, S. G. (2025). u. a. Wearable Monitoring Captures Sleep Disturbances in Patients With Chronic Inflammatory Demyelinating Polyneuropathy. *J Peripher Nerv Syst Dezember*, *30*(4), e70069. 10.1111/jns.70069 10.1111/jns.70069 PMC12536332 41257389

[24] Gashi, S., Oldrati, P., Moebus, M., Hilty, M., Barrios, L., Ozdemir, F. (2024). Modeling multiple sclerosis using mobile and wearable sensor data. NPJ Digit Med. 20240311. Aufl. 11. März ;7(1):64. Located at: PubMed-not-MEDLINE; 38467710. 10.1038/s41746-024-01025-8 10.1038/s41746-024-01025-8 PMC10928076 38467710

[25] Block, V. J., Lizée, A., Crabtree-Hartman, E., Bevan, C. J., Graves, J. S., & Bove, R. (2017). u. a. Continuous daily assessment of multiple sclerosis disability using remote step count monitoring. *J Neurol Februar*, *264*(2), 316–326. 10.1007/s00415-016-8334-6 10.1007/s00415-016-8334-6 PMC5292081 27896433

[26] Thompson, A. J., Banwell, B. L., Barkhof, F., Carroll, W. M., Coetzee, T., & Comi, G. (2018). u. a. Diagnosis of multiple sclerosis: 2017 revisions of the McDonald criteria. *Lancet Neurol Februar*, *17*(2), 162–173. 10.1016/S1474-4422(17)30470-2 10.1016/S1474-4422(17)30470-2 29275977

[27] Piercy, K. L., Troiano, R. P., Ballard, R. M., Carlson, S. A., Fulton, J. E., & Galuska, D. A. (2018). u. a. The Physical Activity Guidelines for Americans. *JAMA 20 November*, *320*(19), 2020. 10.1001/jama.2018.14854 10.1001/jama.2018.14854 PMC9582631 30418471

[28] Herrmann, S. D., Willis, E. A., Ainsworth, B. E., Barreira, T. V., Hastert, M., & Kracht, C. L. (2024). u. a. Adult Compendium of Physical Activities: A third update of the energy costs of human activities. J Sport Health Sci. Januar 2024;13(1):6–12. 10.1016/j.jshs.2023.10.010 10.1016/j.jshs.2023.10.010 PMC10818145 38242596

[29] R Core Team. R: A Language and Environment for Statistical Computing [Internet]. Vienna, Austria: R Foundation for Statistical Computing (2024). Verfügbar unter: https://www.R-project.org

[30] Wickham, H. (2016). ggplot2: Elegant Graphics for Data Analysis [Internet]. Cham: Springer International Publishing; [zitiert 11. Oktober 2025]. (Use R!). Verfügbar unter: http://link.springer.com/10.1007/978-3-319-24277-4 doi:10.1007/978-3-319-24277-4.

[31] Wickham, H., Averick, M., Bryan, J., Chang, W., McGowan, L., & François, R. (2019). u. a. Welcome to the Tidyverse. *J Open Source Softw 21 November*, *4*(43), 1686. 10.21105/joss.01686

[32] HarrellJr, F. E., Cole, B., & Dupont, C. (2025). Hmisc: Harrell Miscellaneous [Internet]. R Foundation for Statistical Computing; Verfügbar unter: https://hbiostat.org/R/Hmisc/10.32614/CRAN.package.Hmisc

[33] Berger, W., Payne, M. W. C., & Morrow, S. A. (2017). Self-reported maximum walking distance in persons with MS may affect the EDSS. *J Neurol Sci August*, *379*, 77–80. 10.1016/j.jns.2017.05.035 10.1016/j.jns.2017.05.035 28716284

[34] Dalla-Costa, G., Radaelli, M., Maida, S., Sangalli, F., Colombo, B., & Moiola, L. (2017). u. a. Smart watch, smarter EDSS: Improving disability assessment in multiple sclerosis clinical practice. *J Neurol Sci Dezember*, *383*, 166–168. 10.1016/j.jns.2017.10.043 10.1016/j.jns.2017.10.043 29246607

[35] Chorschew, A., Kesgin, F., Bellmann-Strobl, J., Flachenecker, P., Schiffmann, I., & Rosenthal, F. (2023). u. a. Translation and validation of the multiple sclerosis walking scale 12 for the German population – the MSWS-12/D. *Health Qual Life Outcomes 9 Oktober*, *21*(1), 110. 10.1186/s12955-023-02190-2 10.1186/s12955-023-02190-2 PMC10563229 37814258

[36] Engelhard, M. M., Patek, S. D., Lach, J. C., & Goldman, M. D. (2018). Real-world walking in multiple sclerosis: Separating capacity from behavior. *Gait Posture Januar*, *59*, 211–216. 10.1016/j.gaitpost.2017.10.015 10.1016/j.gaitpost.2017.10.015 PMC5695705 29078135

[37] Goldman, M. D., Marrie, R. A., & Cohen, J. A. (2008). Evaluation of the six-minute walk in multiple sclerosis subjects and healthy controls. *Mult Scler J April*, *14*(3), 383–390. 10.1177/1352458507082607 10.1177/1352458507082607 17942508

[38] Stellmann, J. P., Neuhaus, A., Götze, N., Briken, S., Lederer, C., & Schimpl, M. (2015). April, u. a. Ecological Validity of Walking Capacity Tests in Multiple Sclerosis. Reindl M, Herausgeber. PLOS ONE. 16. ;10(4):e0123822. 10.1371/journal.pone.0123822 10.1371/journal.pone.0123822 PMC4399985 25879750

[39] Hoogervorst, E. L. J. (2003). One year changes in disability in multiple sclerosis: neurological examination compared with patient self report. *J Neurol Neurosurg Psychiatry 1 April*, *74*(4), 439–442. 10.1136/jnnp.74.4.439 10.1136/jnnp.74.4.439 PMC1738360 12640058

[40] Fjeldstad, C., Fjeldstad, A. S., & Pardo, G. (2015). Use of Accelerometers to Measure Real-Life Physical Activity in Ambulatory Individuals with Multiple Sclerosis. *Int J MS Care 1 September*, *17*(5), 215–220. 10.7224/1537-2073.2014-037 10.7224/1537-2073.2014-037 PMC4599358 26472942

[41] Klassen, L., Schachter, C., & Scudds, R. (2008). An exploratory study of two measures of free-living physical activity for people with multiple sclerosis. *Clin Rehabil März*, *22*(3), 260–271. 10.1177/0269215507082740 10.1177/0269215507082740 18285434

[42] Woelfle, T., Bourguignon, L., Lorscheider, J., Kappos, L., Naegelin, Y., & Jutzeler, C. R. (2023). Wearable Sensor Technologies to Assess Motor Functions in People With Multiple Sclerosis: Systematic Scoping Review and Perspective. *J Med Internet Res 27 Juli*, *25*, e44428. 10.2196/44428 10.2196/44428 PMC10415952 37498655

[43] Kratz, A. L., Murphy, S. L., & Braley, T. J. (2017). Ecological Momentary Assessment of Pain, Fatigue, Depressive, and Cognitive Symptoms Reveals Significant Daily Variability in Multiple Sclerosis. *Arch Phys Med Rehabil November*, *98*(11), 2142–2150. 10.1016/j.apmr.2017.07.002 10.1016/j.apmr.2017.07.002 PMC5660933 28729168

[44] Powell, D. J. H., Liossi, C., Schlotz, W., & Moss-Morris, R. (2017). Tracking daily fatigue fluctuations in multiple sclerosis: ecological momentary assessment provides unique insights. *J Behav Med Oktober*, *40*(5), 772–783. 10.1007/s10865-017-9840-4 10.1007/s10865-017-9840-4 PMC5613039 28281106

[45] Petek, B. J., Al-Alusi, M. A., Moulson, N., Grant, A. J., Besson, C., & Guseh, J. S. (2023). u. a. Consumer Wearable Health and Fitness Technology in Cardiovascular Medicine. *J Am Coll Cardiol Juli*, *82*(3), 245–264. 10.1016/j.jacc.2023.04.054 10.1016/j.jacc.2023.04.054 PMC10662962 37438010

[46] Goldsack, J. C., Coravos, A., Bakker, J. P., Bent, B., Dowling, A. V., & Fitzer-Attas, C. (2020). u. a. Verification, analytical validation, and clinical validation (V3): the foundation of determining fit-for-purpose for Biometric Monitoring Technologies (BioMeTs). *Npj Digit Med 14 April*, *3*(1), 55. 10.1038/s41746-020-0260-4 10.1038/s41746-020-0260-4 PMC7156507 32337371

[47] Tedesco, S., Sica, M., Ancillao, A., Timmons, S., Barton, J., & O’Flynn, B. (2019). Mai. Accuracy of consumer-level and research-grade activity trackers in ambulatory settings in older adults. Najafi B, Herausgeber. PLOS ONE. 21. ;14(5):e0216891. 10.1371/journal.pone.0216891 10.1371/journal.pone.0216891 PMC6529154 31112585

[48] Ferguson, T., Rowlands, A. V., Olds, T., & Maher, C. (2015). The validity of consumer-level, activity monitors in healthy adults worn in free-living conditions: a cross-sectional study. *Int J Behav Nutr Phys Act Dezember*, *12*(1), 42. 10.1186/s12966-015-0201-9 10.1186/s12966-015-0201-9 PMC4416251 25890168

[49] Wahl, Y., Düking, P., Droszez, A., Wahl, P., & Mester, J. (2017). Criterion-Validity of Commercially Available Physical Activity Tracker to Estimate Step Count, Covered Distance and Energy Expenditure during Sports Conditions. *Front Physiol 22 September*, *8*, 725. 10.3389/fphys.2017.00725 10.3389/fphys.2017.00725 PMC5615304 29018355

[50] Epping, P. Z., Hagler, R., Werner, N. M., Voth, J., Schmidt, L., & Huntemann, N. (2026). u. a. Case-Based Lessons on Remote Patient Monitoring in Neurology Using Consumer-Grade Wearables. *J Cent Nerv Syst Dis 11 Februar*, *18*, 11795735261419641. 10.1177/11795735261419641 10.1177/11795735261419641 PMC12894642 41695242

[51] Hug, A., Spingler, T., Pleines, V., Heutehaus, L., Schoenfeld, M. A., & Hauptmann, B. (2025). u. a. Exploring the relationship of clinical walking tests with 8-months inertial measurement unit (IMU)-based real world mobility tracking in stroke and spinal cord injury survivors. *Neurol Res Pract 9 Mai*, *7*(1), 30. 10.1186/s42466-025-00386-z 10.1186/s42466-025-00386-z PMC12063441 40341076

[52] Holland, A. E., Spruit, M. A., Troosters, T., Puhan, M. A., Pepin, V., & Saey, D. (2014). u. a. An official European Respiratory Society/American Thoracic Society technical standard: field walking tests in chronic respiratory disease. *Eur Respir J Dezember*, *44*(6), 1428–1446. 10.1183/09031936.00150314 10.1183/09031936.00150314 25359355

[53] Moore, J. L., Potter, K., Blankshain, K., Kaplan, S. L., O’Dwyer, L. C., & Sullivan, J. E. (2018). A Core Set of Outcome Measures for Adults With Neurologic Conditions Undergoing Rehabilitation: A CLINICAL PRACTICE GUIDELINE. *J Neurol Phys Ther Juli*, *42*(3), 174–220. 10.1097/NPT.0000000000000229 10.1097/NPT.0000000000000229 PMC6023606 29901487

[54] Barry, G., Galna, B., Lord, S., Rochester, L., & Godfrey, A. (2015). Defining ambulatory bouts in free-living activity: Impact of brief stationary periods on bout metrics. *Gait Posture Oktober*, *42*(4), 594–597. 10.1016/j.gaitpost.2015.07.062 10.1016/j.gaitpost.2015.07.062 26299735

