# Supplementary material for: Rethinking EDSS-based ambulation assessment in multiple sclerosis using continuous variable monitoring
Source: Neurol Res Pract. 2026 Jun 12;8(1):47. doi: 10.1186/s42466-026-00501-8 (PMC13263936; doi:10.1186/s42466-026-00501-8)
Supplement: Supplementary file 1 — Supplementary Material 1. [file 42466_2026_501_MOESM1_ESM.pdf]

Supplementary Figure 1

UKD Universitätsklinikum Düsseldorf hhu Heinrich Heine Universität Düsseldorf

Walking Distance - Study

Pat-ID: ____________________

Visit: ____________________

Date: ____________________

Examiner: ____________________

Baseline:

Height: ______________ cm Weight: ______________ kg

First Diagnosis MS: ______________EDSS: ______________

Maximum possible walking distance (Patient´s estimate):____________meter

Maximum possible steps (Patient´s estimate): ______________

Testing:

Start (time): ____________(hh:mm:ss) End (time):___________ (hh:mm:ss)

Distance walked (Meter): ______________

Steps walked (number):______________

Other:

Comment Nurse/Physician:

____

Comments Technology/ Reason for not wearing SmartWatch:

____

Protocol Version 1.0 PP2024 translated into English

Page 1 von 1

Supplementary Figure 1. Questionnaire for Walking Distance Assessment.
Baseline data, including height (cm), weight (kg), current Expanded Disability Status Scale (EDSS), and initial diagnosis of multiple sclerosis (MS), were recorded prior to the test. Patients were asked to estimate the distance they could walk continuously (meters) and the corresponding number of steps. Investigators noted the start and end times of the walking task. The actual distance walked (meters) and the number of steps were recorded at the end. A large field was provided for additional comments during the assessment.

Supplementary Figure 2

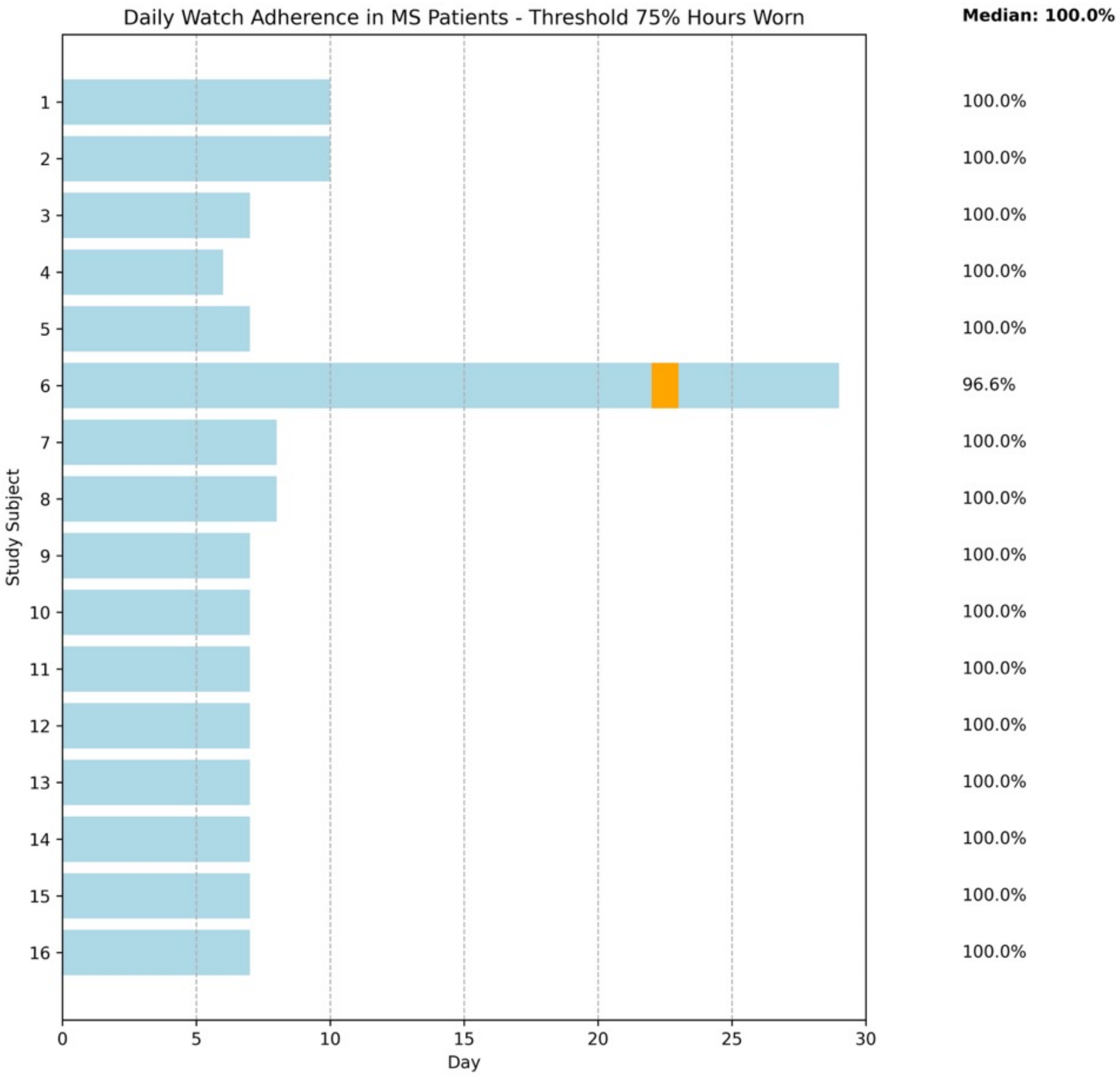

Supplementary Figure 2. Adherence rates (%) per Patient over the Study Period (n = 16).
Blue bars represent device wearing time, while orange bars indicate non-wearing intervals. The x-axis shows study days, and the y-axis depicts individual patients.

Supplementary Figure 3

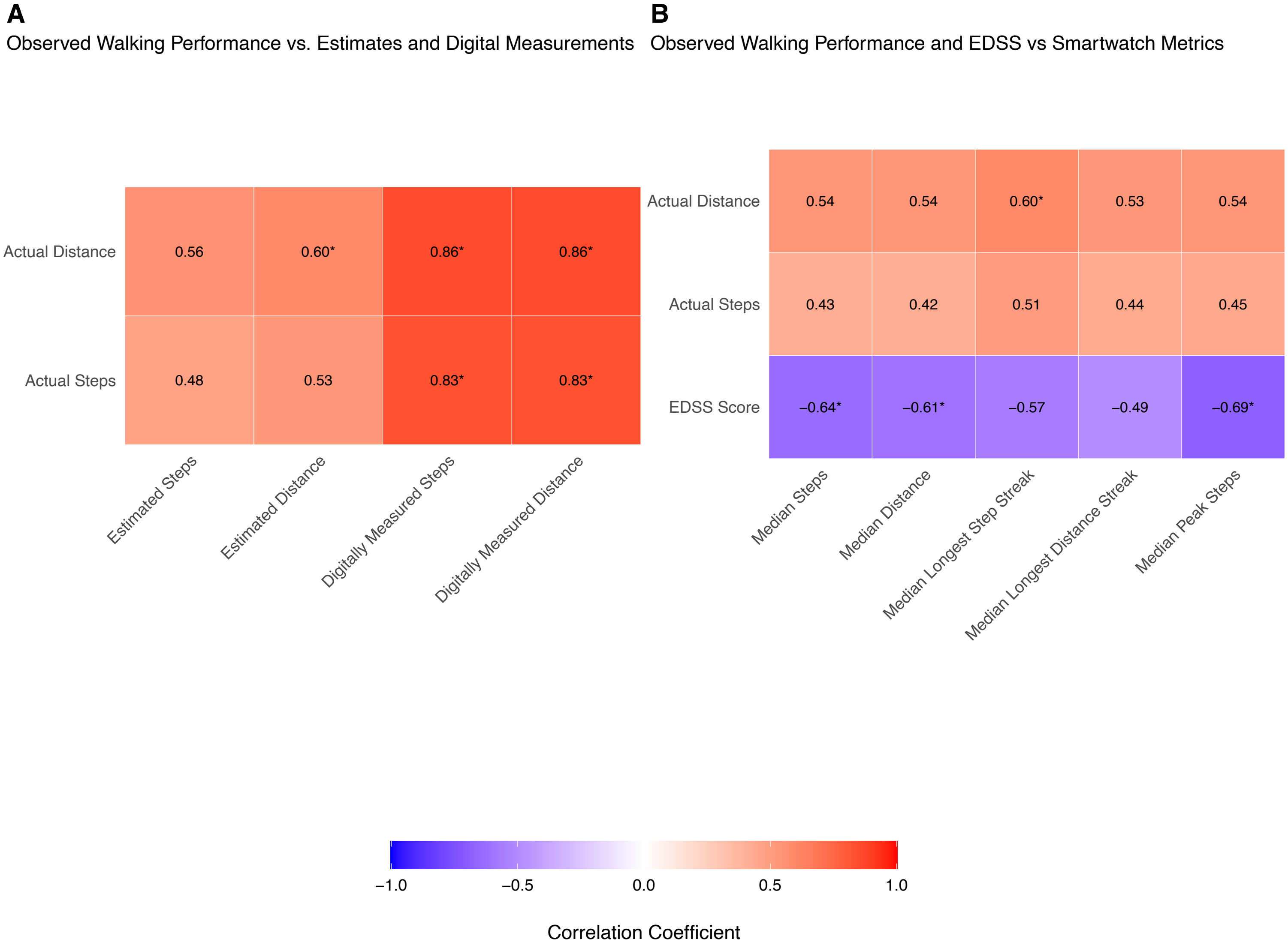

Supplementary Figure 3. Correlation Heatmaps of Observed Walking Performance, Digital Measurements, and Disability Scores with FDR Correction.
Correlation between observed walking performance (actual distance and steps during the walking test) and patient-reported estimates, as well as digitally measured steps and distance. (B) Correlation between observed walking performance and EDSS scores with smartwatch-derived activity parameters, including median steps, median distance, longest step and distance streaks, and peak steps. Red indicates positive correlations, while blue indicates negative correlations. Asterisks denote statistically significant correlations after FDR correction (*q < 0.05). Pearson or Spearman correlation coefficients were applied depending on data distribution.

Abbreviations: EDSS, Expanded Disability Status Scale

Supplementary Figure 4

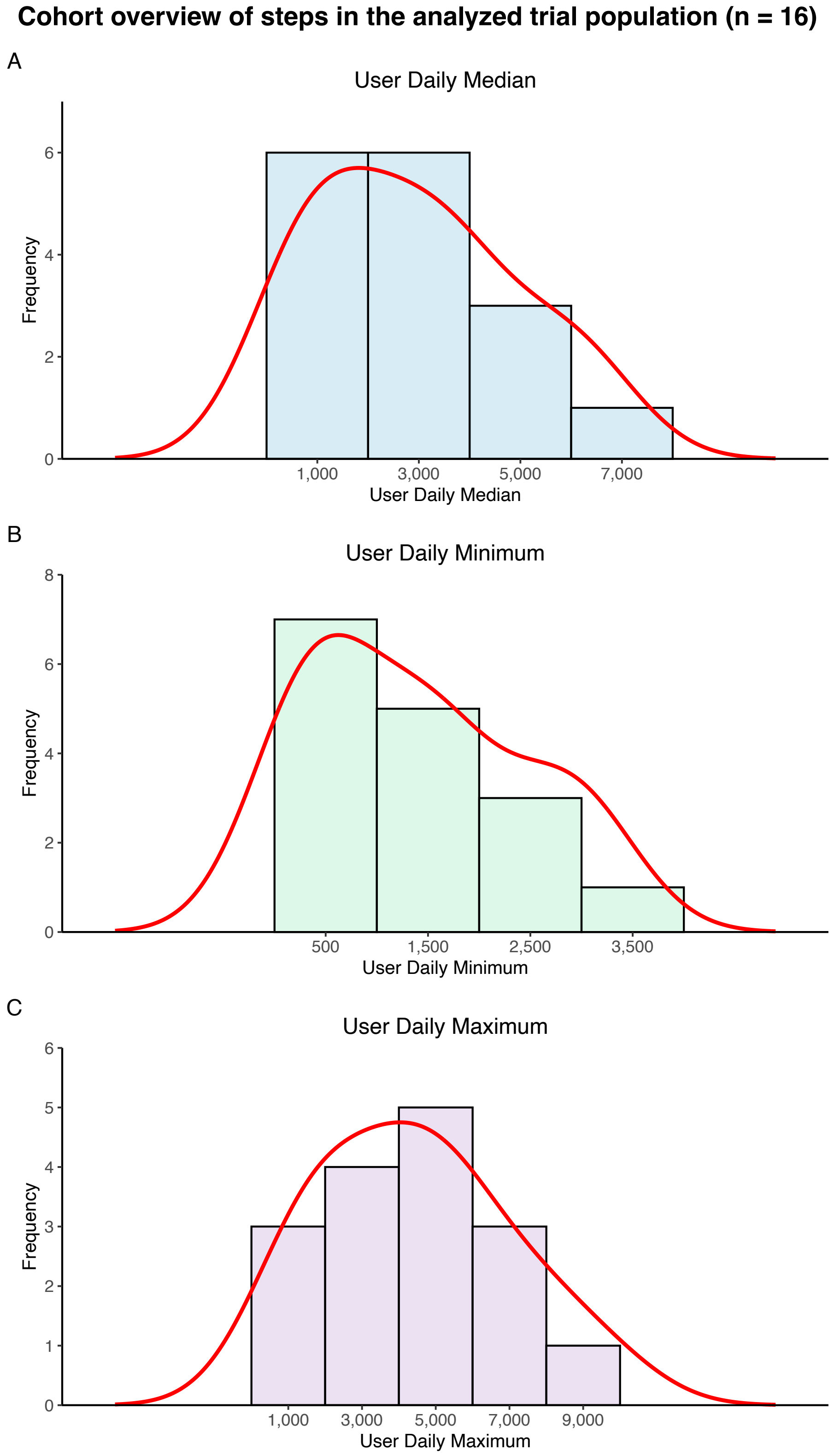

Supplementary Figure 4. Cohort Overview of Daily Step Counts in the Analyzed Trial Population (n = 16). Histograms show the distribution of daily steps measured via smartwatch, with density curves overlaid in red. Panel (A) depicts the median daily steps per user, panel (B) depicts the minimum daily steps, and panel (C) depicts the maximum daily steps. The x-axis represents steps per day, and the y-axis represents frequency (number of users). Histogram bars represent ranges of daily step counts, with x-axis labels indicating the central value of each range. Panel labels (A–C) are positioned at the top left of each plot.

Supplementary Table 1

		Overall
***n* (%)**		**16 (100.0)**
Study center, *n* (%)	DD	8 (50.00)
	SC	8 (50.00)
Sex, *n* (%)	w	11 (68.75)
	m	5 (31.25)
Age at inclusion [y], median [Q1, Q3]		57.5 [49.25, 63.25]
Age at diagnosis [y], median [Q1, Q3]		42 [27.50, 56.25]
Disease duration at inclusion [y], median [Q1, Q3]		12.5 [3.50, 22.00]
Baseline EDSS, median [Q1, Q3]		4.5 [3.0, 6.0]
BMI (kg/m^2), median [Q1, Q3]		26.37 [22.95, 29.96]
Treatment, *n (%)*		16 [100.0]
Anti CD-20, *n (%)*		11 [100.0]
Teriflunomid, *n (%)*		1 [100.0]
No Specific Treatment, *n (%)*		4 [100.0]
Duration in study [d], median [Q1, Q3]		7 [7, 10]
V1 Steps (n) in Walking Distance Test, median [Q1, Q3]		443 [229.50, 1255.50]
V1 Walking Distance (m) in Walking Distance Test, median [Q1, Q3]		214.33 [111.80, 694.58]
V2 Steps (n) in Walking Distance Test, median [Q1, Q3]		495.5 [326.00, 700.50]

Supplementary Table 1

		Overall
n **(%)**		**16 (100.0)**
V2 Walking Distance (m) in Walking Distance Test, median [Q1, Q3]		250.69 [177.63, 369.81]
Average steps per day, mean (SD)		2698.15 (2119.66)
Median steps per day, median [Q1, Q3]		2210 [796.50, 4118.50]
Maximum steps per day per subject, mean (SD)		4444.81(2616.73)
Minimum steps per day per subject, mean (SD)		1381.25 (1036.55)
Average Distance per day, mean (SD)		2031.81 (1606.78)
Median Distance per day, median [Q1, Q3]		1779.8 [590.30,3136.80]
Maximum Distance per day per subject, mean (SD)		3436.71 (1957.71)
Minimum Distance per day per subject, mean (SD)		1017.77 (771.09)
Median Peak Steps per day, median [Q1, Q3]		564 [254.50,984.25]

Explanation:
n = quantity
SC = Sauerlandklinik Hachen
DD = University Hospital Düsseldorf
w = women
m = men
y = year
V1 = visit 1 (Start-of-study visit)
V2= visit 2 (End-of-study-visit)
SD = standard deviation
Q1 = lower quartile
Q3 = upper quartile
BMI = body mass index
EDSS = Expanded Disability Status Scale

Supplementary Table 1. Cohort Characteristics.

Supplementary Table 2

Variable1	Variable2	Coefficient	p-Value	Correlation Method	Significance	FDR (q-Value)	significance_FDR
V1_Steps	V1_Estimate_Steps	0.48	0.0598	Spearman		0.0734	
V1_Steps	V1_Estimate_Distance	0.53	0.0333	Spearman	*	0.0559	
V1_Steps	V1_Steps_Digital	0.83	<0.001	Spearman	*	<0.001	*
V1_Steps	V1_Distance_Digital	0.83	<0.001	Spearman	*	<0.001	*
V1_Distance	V1_Estimate_Steps	0.56	0.0246	Spearman	*	0.0554	
V1_Distance	V1_Estimate_Distance	0.60	0.0134	Spearman	*	0.0448	*
V1_Distance	V1_Steps_Digital	0.86	<0.001	Spearman	*	<0.001	*
V1_Distance	V1_Distance_Digital	0.86	<0.001	Spearman	*	<0.001	*
V1_Steps	median_longest_step_streak_15	0.51	0.0464	Spearman	*	0.0627	
V1_Steps	median_longest_distance_streak_15	0.44	0.0866	Spearman		0.0936	
V1_Distance	median_longest_step_streak_15	0.60	0.0166	Spearman	*	0.0448	*
V1_Distance	median_longest_distance_streak_15	0.53	0.0373	Spearman	*	0.0559	
V1_Steps	median_longest_step_streak_15	0.51	0.0464	Spearman	*	0.0627	
V1_Steps	median_longest_distance_streak_15	0.44	0.0866	Spearman		0.0936	
V1_Steps	Smartwatch_Intervall_peak_steps_median	0.45	0.0822	Spearman		0.0936	
V1_Steps	Median_Steps_SmartwatchIntervall	0.43	0.1011	Spearman		0.1049	
V1_Steps	Median_Distance_SmartwatchIntervall	0.42	0.1062	Spearman		0.1062	
V1_Distance	median_longest_step_streak_15	0.60	0.0166	Spearman	*	0.0448	*
V1_Distance	median_longest_distance_streak_15	0.53	0.0373	Spearman	*	0.0559	
V1_Distance	Smartwatch_Intervall_peak_steps_median	0.54	0.0316	Spearman	*	0.0559	
V1_Distance	Median_Steps_SmartwatchIntervall	0.54	0.0316	Spearman	*	0.0559	
V1_Distance	Median_Distance_SmartwatchIntervall	0.54	0.0338	Spearman	*	0.0559	
EDSS_V1	median_longest_step_streak_15	-0.57	0.0224	Spearman	*	0.0550	
EDSS_V1	median_longest_distance_streak_15	-0.49	0.0517	Spearman		0.0665	
EDSS_V1	Smartwatch_Intervall_peak_steps_median	-0.69	0.0032	Spearman	*	0.0170	*
EDSS_V1	Median_Steps_SmartwatchIntervall	-0.64	0.0082	Spearman	*	0.0367	*
EDSS_V1	Median_Distance_SmartwatchIntervall	-0.61	0.0123	Spearman	*	0.0448	*

Supplementary Table 2. Correlations between walking-related Measures at Visit 1 (V1) and Clinical and Digital Outcomes.
The table presents paired variables, correlation coefficients, p-values, FDR q-values, correlation methods (Pearson or Spearman), and significance levels (* indicates statistical significance for nominal ($p < 0.05$) or FDR-adjusted ($q < 0.05$) values). Positive coefficients indicate direct associations, whereas negative coefficients indicate inverse associations.
Abbreviations:
V1_Steps refers to actual steps at visit 1; V1_Distance to the actual distance walked at visit 1 in meters; V1_Estimate_Steps and V1_Estimate_Distance to patient-estimated steps and distance; V1_Steps_Digital and V1_Distance_Digital to digitally recorded steps and distance at visit 1; median_longest_step_streak_15 and median_longest_distance_streak_15 to the median longest step or distance streak over the smartwatch interval; Smartwatch_Intervall_peak_steps_median, Median_Steps_SmartwatchIntervall, and Median_Distance_SmartwatchIntervall to smartwatch derived step and distance metrics; and EDSS_V1 to the Expanded Disability Status Scale at visit 1.

Supplementary Table 3

Patient	Estimated	Actual	Difference
Patient 1	100	672.28	572.28
Patient 2	200	742.16	542.16
Patient 3	500	348.84	-151.16
Patient 4	500	56.64	-443.36
Patient 5	700	276.7	-423.3
Patient 6	10	234.28	224.28
Patient 7	500	174.36	-325.64
Patient 8	5	8.36	3.36
Patient 9	300	135.7	-164.3
Patient 10	2000	1489.1	-510.9
Patient 11	400	100.16	-299.84
Patient 12	1500	716.88	-783.12
Patient 13	25	123.44	98.44
Patient 14	10	10.32	0.32
Patient 15	400	194.38	-205.62
Patient 16	2000	2300	300

Supplementary Table 3. Estimated and Actual Walking Distance (meters) for each Patient (n = 16).
The table presents the estimated and actual walking distances in the respective columns. The "Difference" column represents the deviation between the estimated and actual distances; positive values indicate underestimation by the patient, whereas negative values indicate overestimation.

Supplementary Table 4

Patient	V1_Distance	V2_Distance	Difference
Patient 1	672.28	292.04	-380.24
Patient 2	742.16	247.18	-494.98
Patient 3	348.84	254.2	-94.64
Patient 4	56.64	255	198.36
Patient 5	276.7	197.98	-78.72
Patient 6	234.28	432.72	198.44
Patient 7	174.36	306.9	132.54
Patient 8	8.36	8.3	-0.06
Patient 9	135.7	165.3	29.6
Patient 10	1489.1	667.72	-821.38
Patient 11	100.16	76.12	-24.04
Patient 12	716.88	1118.4	401.52
Patient 13	123.44	189.96	66.52
Patient 14	10.32	0	-10.32
Patient 15	194.38	211.62	17.24
Patient 16	2300	2150	-150

Supplementary Table 4. Walking Distances (meters) for each Patient (n = 16) at Visit 1 (V1) and Visit 2 (V2), with the resulting Difference calculated as V1 minus V2.
Positive values indicate a decrease in distance at V2 compared to V1, while negative values indicate an increase.

Supplementary Table 5

Test	V1 Estimated vs Actual	V2 vs V1
N	16	16
Mean_Abs_Diff	315.5	193.66
Median_Abs_Diff	299.92	113.59
SD_Abs_Diff	218.61	225.92
Shapiro_W	0.968	0.801
Shapiro_p	0.8	0.003
T_test_t	5.773	
T_test_p	p<0.001	
Wilcox_V		136
Wilcox_p		p<0.001

Supplementary Table 5. Summary of statistical Tests on Walking Distance Data.
The first column ("Test") lists the performed analyses: Mean_Abs_Diff and Median_Abs_Diff represent the mean and median absolute differences, and SD_Abs_Diff indicates the standard deviation. Shapiro_W and Shapiro_p report results of the Shapiro–Wilk normality test. Depending on distributional assumptions, either a one-sample t-test (T_test_t, T_test_p) or a one-sample Wilcoxon signed-rank test (Wilcoxon_V, Wilcoxon_p) was applied to assess deviation from zero. The second column presents results for absolute differences between estimated and actual walking distances at visit 1 (V1), while the third column shows results for absolute within-subject differences between visit 2 (V2) and visit 1 (V1).
